# Casein-assisted enhancement of the compressive strength of biocemented sand

**DOI:** 10.1038/s41598-022-16879-9

**Published:** 2022-07-26

**Authors:** Masato Miyake, Daehyun Kim, Toshiro Hata

**Affiliations:** 1 Department of Civil and Environmental Engineering, Hiroshima University, 1-4-1 Kagamiyama, Higashi-hiroshima, Hiroshima, 739-8527 Japan; 2 School of Sustainable Engineering and the Built Environment, Arizona State University, Tempe, USA

**Keywords:** Environmental sciences, Engineering, Materials science

## Abstract

As a soil biomineralization process, casein-assisted enzyme-induced carbonate precipitation (EICP) yielded biocemented specimens with significantly higher compressive strength than specimens cemented by regular or skim-milk-assisted EICP treatments. The compound concentration and curing strategy of casein-assisted EICP were experimentally optimized to maximize the compressive strength of precipitates with low calcium carbonate content. Under the optimized EICP conditions (0.893 M urea, 0.581 M CaCl_2_, 2.6 g/L urease enzyme, and 38.87 g/L casein), the unconfined compressive strengths reached 2 MPa. The scanning electron micrographs of selected samples provided microscopic evidence that EICP treatments assisted using skim milk and casein impart distinctive strength-enhancement mechanisms. The ammonium ions released from urea hydrolysis created an alkaline environment that makes casein dissociated into the pore water. As the casein-containing pore water became more viscous, the increased contact area with particles facilitated the precipitation of co-bound CaCO_3_ minerals and casein in the pore water. Casein was identified as a more efficient assisting agent than skim milk for low-level CaCO_3_ precipitation by EICP treatment.

## Introduction

Biologically induced mineral formation, referred to as biomineralization, is a widely known natural process that modifies the ground condition by precipitating carbonate minerals on the particle surfaces of soil^1–3^. The main consequences of soil biomineralization are improved mechanical stability and reduced hydraulic conductivity of the soil^2,4,5^. Among the engineering applications of biomineralization are slope stabilization^6^, soil liquefaction mitigation^7^, fugitive dust control^8,9^, biocemented soil columns^10,11^, permeability control^12–14^, immobilization of groundwater contaminants^15,16^, enhanced oil recovery^17^, and security improvement of CO_2_ reservoirs^18^.

Enzymatically induced carbonate precipitation (EICP) is one of the well-known biomineralization processes for strengthing of soils, preferably granular material such as sand^8,9,11^. Urea, a source of calcium ions, and urease enzyme are essential substances for EICP treatment and are commonly supplied in an aqueous solution. However, the dissolved casein will increase the liquid viscosity, reducing the percolation speed and sometimes clogging near the inlet side.

Therefore, the applicability of the proposed Casein-based EICP methods will be tested on unclogged samples. The provided urea is hydrolyzed by the enzymatic activity of urease, which generates carbonate ions at sufficiently alkaline pH (8.3 ± 1.0)^19,20^. The carbonate ions released from urea hydrolysis precipitate with the ambient dissolved calcium ions typically provided by calcium chloride in an aqueous solution, forming calcium carbonate crystals with multiple morphologies. The basic biochemical reactions of the EICP process are given below:

1 $${\text{CO}}{({{\text{NH}}}_{2})}_{2(aq)}+3{{\text{H}}}_{2}{\text{O}}\mathop \rightarrow\limits^{Urease}2{{\text{NH}}}_{4(aq)}^{+}+{{\text{HCO}}}_{3(aq)}^{-}+{{\text{OH}}}_{(aq)}^{-}$$

2 $${{\text{HCO}}}_{3(aq)}^{-}\to { {\text{CO}}}_{3(aq)}^{2-}+{{\text{H}}}_{(aq)}^{+}$$

3 $${{\text{Ca}}}_{(aq)}^{2+}+{{\text{CO}}}_{3(aq)}^{2-}\to {\text{CaC{O}}}_{3\left(s\right)}$$

This biomineralization process via urea hydrolysis can also be catalyzed by microorganisms, typically by urease-containing *Sporosarcina pasteurii*. Microorganism-mediated soil mineralization is called microbially induced carbonate precipitation (MICP). However, owing to size incompatibility, microbes cannot readily penetrate pores smaller than medium to fine sand^21^. Biomass accumulation in MICP and bulk mineral deposition in/near the injection zone may cause preferential flows and uneven distributions of substrates and mineral precipitation^22,23^. EICP treatment is considered more approachable than MICP treatment because it removes the efforts of cultivating, monitoring, and maintaining the microorganisms in on-site bioreactors. In addition, the urease enzymes of EICP are smaller than microorganisms (~ 12 nm per subunit versus 0.5–3.0 μm for most microorganisms) and can access finer pores.

In engineering practice, the use of EICP treatment is largely limited by the high price of free urease enzyme^24^. Several approaches for reducing the cost of this enzyme have been proposed. For example, urease has been extracted from various plant sources such as jack bean^25^, jack bean meal^26^, soybean^27^, and watermelon seeds^28^. Recent studies have proposed methodologies that instead increase the precipitation efficiency of the EICP process. Hamdan et al.^29^ proposed a hydrogel-assisted EICP treatment. They interpreted that hydrogel generates a viscous solution that reduces migration of the reactive solution, retaining the reactive solution in the pore space and thus extending the reaction time and increasing the precipitation efficiency. Recently, Almajed et al.^8^ reported an EICP treatment with a modified solution including non-fat milk powder. They found a significant increase in the mechanical strength of the precipitate, which they attributed to the larger calcite crystals at the inter-particle contacts than in past studies using milk powder. In the early stage of EICP research, Nemati and Voordouw^13^ added skim-milk powder as a simple stabilizer to the EICP reactive solution. Almajed et al.^8^ interpreted that milk powder inhibits the reaction by restricting the number of active sites on the enzyme. They assumed that casein in the milk acts as a chelating agent. Other research also focused on casein itself as a soil strengthening binder^30,31^. In these studies, alkaline solutions were artificially prepared by dissolving other substances such as calcium hydroxide^31^ or sodium hydroxide^30^ as casein is soluble in alkaline water conditions.

We hypothesized that casein is the major component in milk powder and plays a key role in creating bigger crystals at the particle contact points during the modified EICP treatment. In this study, we optimize a casein-assisted EICP treatment strategy to enhance the compressive strength of the sand specimens. We experimentally compare the unconfined compressive strengths (UCS) of EICP-treated specimens assisted by skim milk and casein. Scanning electron microscope (SEM) images identified the distinctive strength-enhancement mechanisms of skim milk- and casein-assisted EICP treatments at the microscopic level.

## Results and discussion

### Optimization studies of casein-assisted EICP biocementation

**Figure 1:**
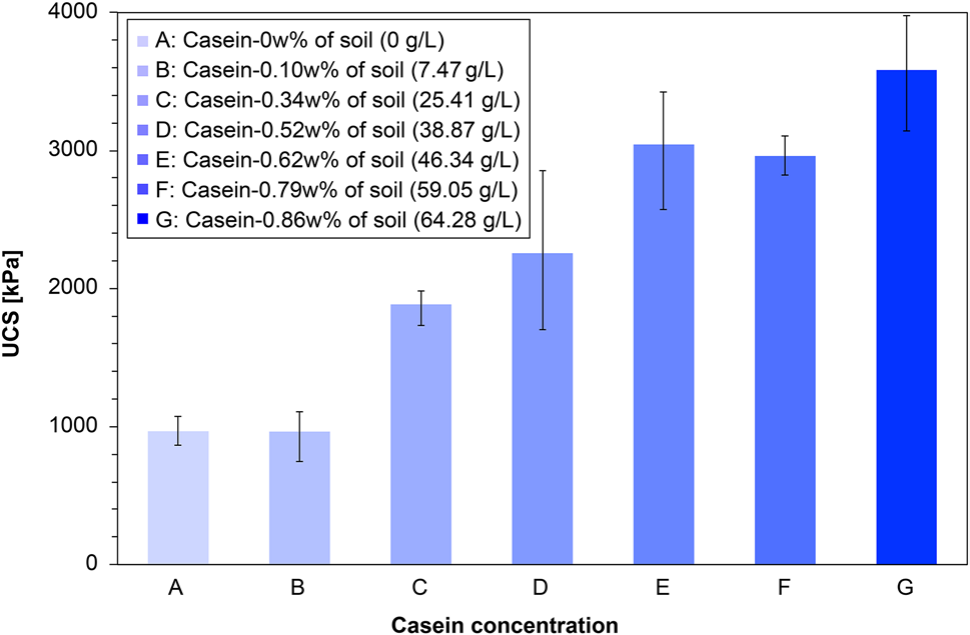
UCS results of specimens treated with varying casein contents and constant EICP-compound concentration.

**Figure 2:**
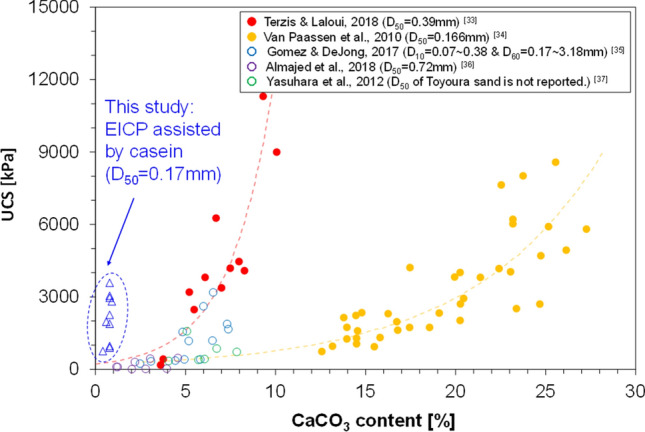
Unconfined compressive strength (USC) versus calcium carbonate (CaCO_3_) content in the specimens treated using biocementation in the literature and using EICP in this study.

Figure 1 shows the UCS measurements of the EICP-treated specimens assisted by casein at different concentrations (the urea/CaCl_2_ concentration was fixed at 0.893 M/0.581 M). In general, increasing the casein content increased the mean UCS strength of the specimens. In the regularly EICP-treated specimens without casein (Case A), the UCS reached almost 1 MPa. In previous studies, a UCS of 1 MPa was scarcely achieved at low CaCO_3_ contents (see Fig. 2)^32–36^. The partially saturated condition might contribute to this surprisingly enhanced compressive strength by concentrating a large portion of the pore water at the particle-contact sites and creating concave menisci between the particles^37^. In this case, more solutes and enzymes are distributed along with the localized pore water, thus concentrating the CaCO_3_ precipitation at the particle contacts. In Case B with a casein content of 7.47 g/L, the mean UCS strength was not noticeably improved from that of Case A without casein, implying that the casein content was insufficient to support effective CaCO_3_ and local-particle bonding. In Case G with a casein content of 64.28 g/L, the mean UCS strength was definitely increased, indicating that the ammonium ions (${\mathrm{NH}}_{4}^{+}$) released from hydrolysis of 0.893 M of urea with 2.6 g/L of enzyme created a sufficiently alkaline environment for casein dissolution into the pore water. The fluctuation of mean UCS strength around Case E (with 46.34 g/L casein) implies that the casein dissolution stagnated above some threshold casein content. As this study was not intended to maximize the compressive strength, we selected 38.87 g/L (Case D) as the casein content in the EICP treatment with a urea/CaCl_2_ concentration of 0.893 M/0.581 M for subsequent studies.

**Figure 3:**
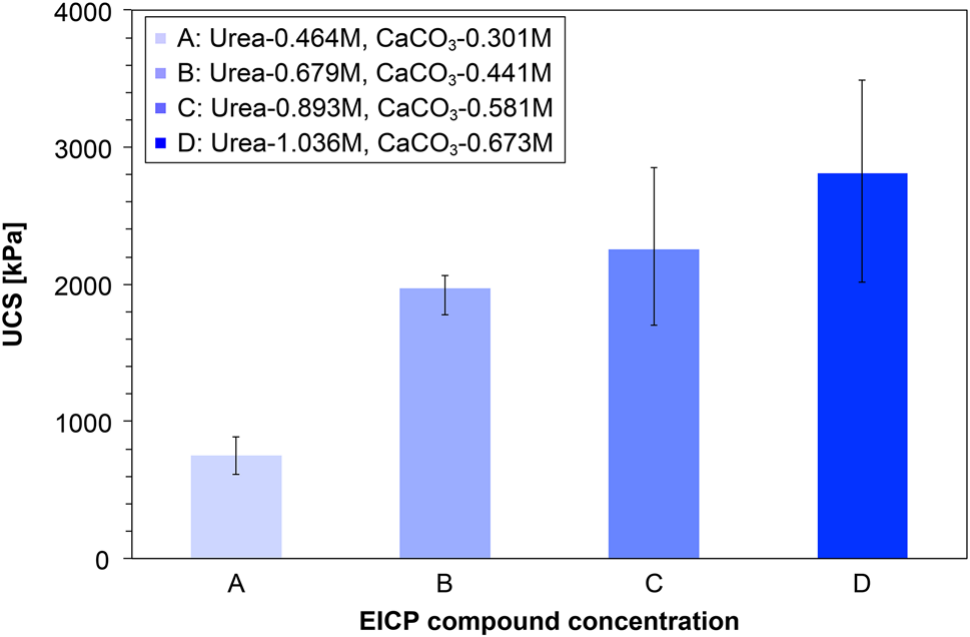
UCS results of the specimens treated with varying EICP-compound concentrations and constant casein content.

Figure 3 shows the UCS results after optimizing the EICP concentration while maintaining the casein content at 38.87 g/L. Overall, the mean UCS increased after increasing the amounts of urea and CaCl_2_ in the EICP treatment, indicating that casein did not negatively affect the CaCO_3_ precipitation via EICP with ultra-low urea/CaCl_2_ concentrations (below 1.036 M/0.673 M). In Case A (urea/CaCl_2_ concentration = 0.464 M/0.301 M), the UCS reached 752 kPa. Considering the theoretical target of the precipitation content in Case A (up to 0.4 wt% of soil), a compressive strength above 700 kPa was significantly enhanced from the UCS results of former biocementation-related research (see Fig. 2). In this case, we cannot definitively state that CaCO_3_ precipitation mainly contributes to strength enhancement. However, the urea content (0.464 M) might have been sufficient to create the requisite alkaline conditions for casein dissolution via urea hydrolysis, which produces ammonium ions. The dissolved casein in the pore water was eventually precipitated and provided sparse particle bondings, which improved the compressive strength with relatively small amounts of CaCO_3_. In Case B (urea/CaCl_2_ concentration = 0.679 M/0.441 M), the UCS strength was dramatically increased (by more than 260%) from that of Case A. It was inferred that more urea provides more ammonium ions through urea hydrolysis, easily creating the alkaline environment for casein dissolution. Pore water containing dissolved casein becomes viscous and remains near the particle contacts. The dominant strengthening mechanism in Case B is the combined binding effect of CaCO_3_ precipitation and casein coagulation. Through this combined particle-binding mechanism, the mean UCS strength gradually increased as the urea/CaCl_2_ content increased up to 1.036 M/0.673 M under the casein content-controlled condition. In engineering applications of casein-assisted EICP treatment, the urea/CaCl_2_ concentration can be adjusted to meet the desired mechanical performance or available budget. In the present study, the EICP-compound concentrations in Case C were selected for the EICP containing 38.87 g/L of casein.

### Curing and drying methods for casein-assisted EICP treatment

**Figure 4:**
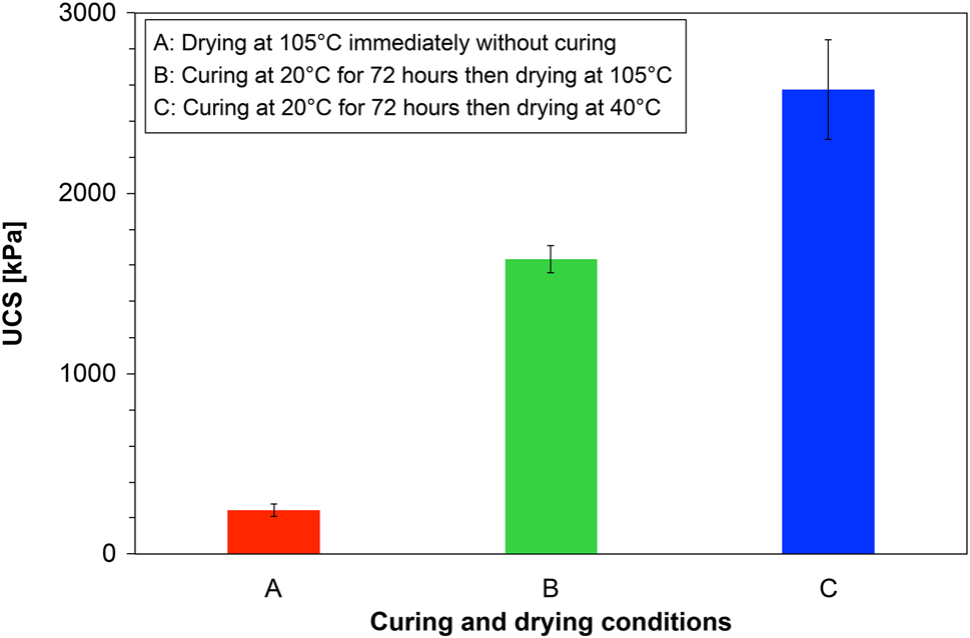
UCS results of the specimens under different curing and drying conditions.

Figure 4 shows the UCS measurements of the casein-assisted EICP-treated specimens under different curing and drying conditions. After instant oven-drying at 105 °C (Case A), the mean UCS strength was 243 kPa, implying a limited biocementation process. The high drying temperature might have deactivated the urease enzyme that catalyzes urea hydrolysis. The pH of the ambient pore water remained nearly neutral or lowly alkaline, inhibiting casein dissolution into the pore water. When the specimens were cured at room temperature and dried at 40 °C for a sufficiently long time (Case C), the mean UCS strength was 2576 kPa, a 158% improvement over that of Case B (1633 kPa) with the same curing period as Case C but a much shorter drying period at 105 °C. This result indicates that urea hydrolysis continued during drying at 40 °C, generating more precipitation of CaCO_3_ minerals and more dissolution of casein in the alkaline environment of the pore water than in case B. Therefore, Case C was selected as the common curing and drying strategy.

### Comparison study of biocemented specimens assisted by skim milk and casein

**Figure 5:**
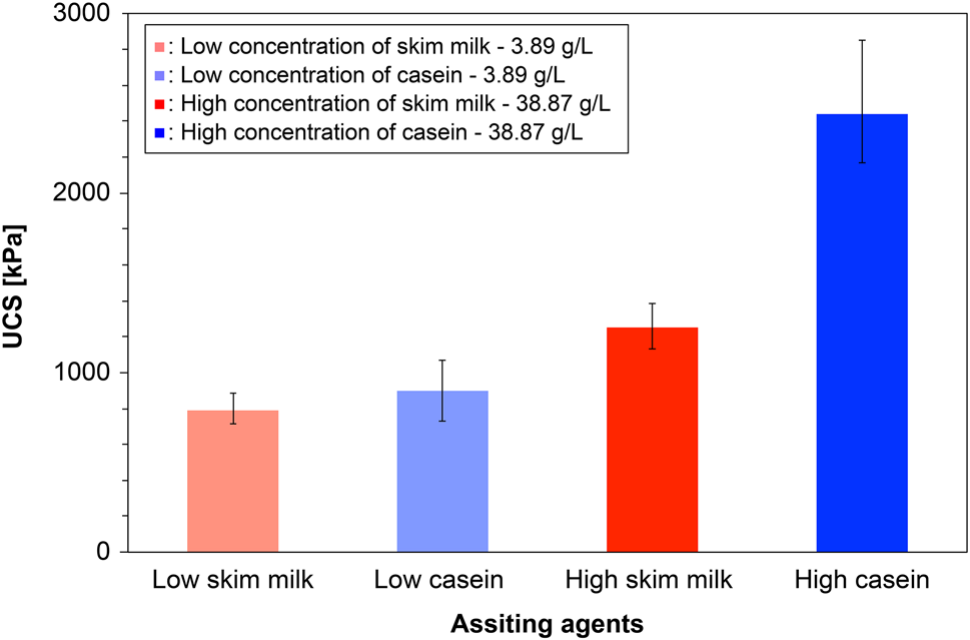
UCS results of the ECIP-treated specimens assisted using the low- and high-concentrations of skim milk and casein.

**Figure 6:**
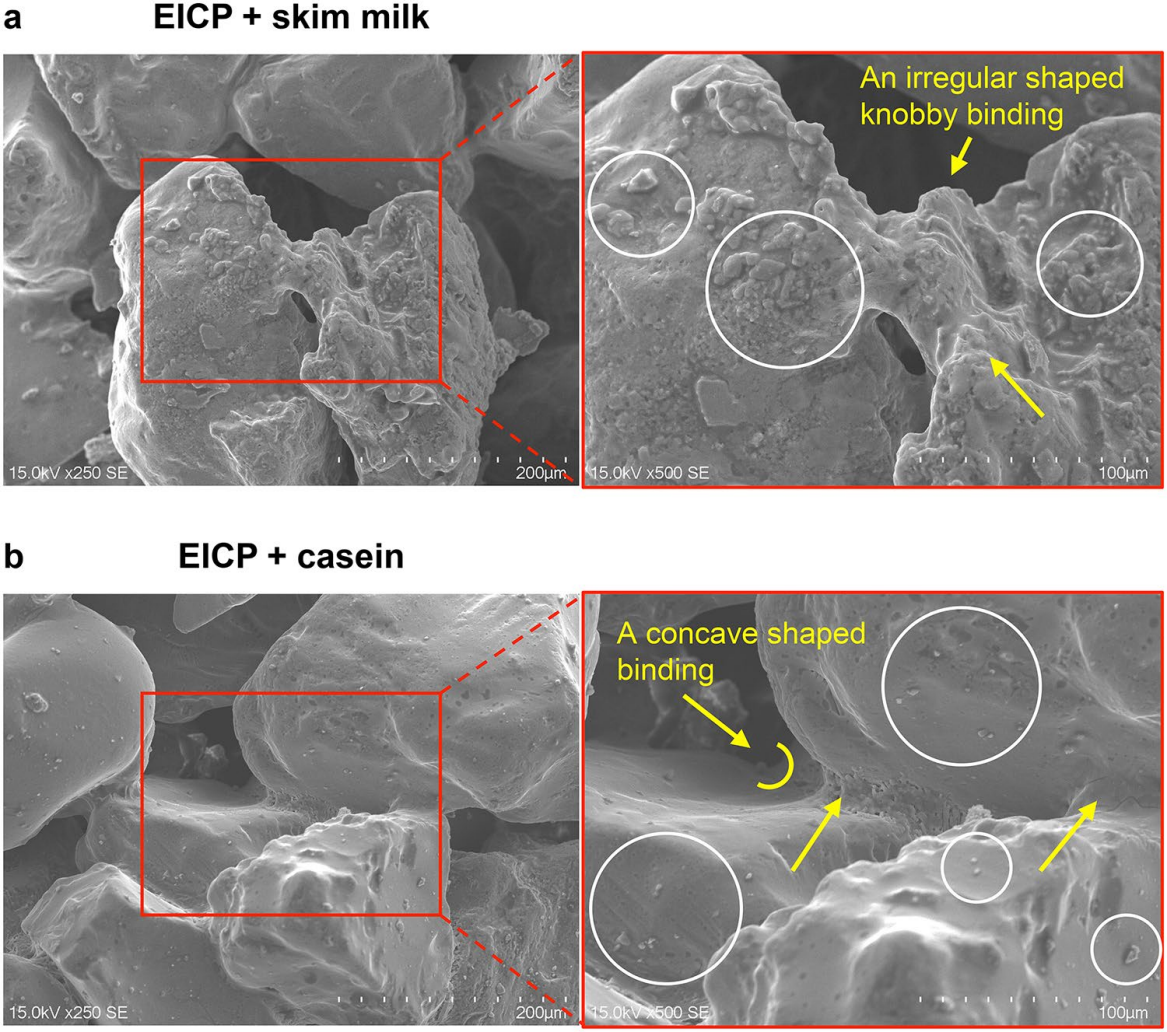
SEM images of the EICP-biocemented specimens assisted using skim milk (**a**) and casein (**b**).

Figure 5 compares the UCS results of the EICP-treated specimens assisted by low and high concentrations of skim milk and casein. When skim milk and casein were added at 3.89 g/L, the mean UCS strengths were 789 and 898, kPa, respectively. When the concentration of skim milk and casein increased to 38.87 g/L, the mean UCS strengths increased to 1251 and 2439 kPa, respectively. Regardless of concentration, the UCS measurements were higher in the casein-assisted EICP treatment than in the milk-assisted EICP treatment (c.f. pale and vivid blue bars in Fig. 5). Therefore, casein was a more efficient enhancing agent of EICP treatment than milk powder, especially when more agents were included. To better understand the distinctive strength-enhancement mechanism, the microscale morphology was visualized by SEM imaging. Figure 6 represents SEM images of selected parts of the test specimens prepared with high additive concentrations. On the skim-milk-assisted EICP-treated sample (Fig. 6a), CaCO_3_ precipitations were agglomerated over the particle surfaces and near the particle contacts. A chunk of crystals formed a knobbly bridge connecting two large sand particles (pointed by yellow arrows in Fig. 6a), which might majorly contribute to the mechanical-strength enhancement of the treated specimens. Moreover, variously sized CaCO_3_ crystals shrouded the sand-particle surface (marked by white-edged circles in Fig. 6a). When casein was added to the EICP compound, the crystals were smaller and more sparsely precipitated on the sand-particle surfaces (marked by white-edged circles in Fig. 6b) and the precipitation was concentrated at the sand-particle contacts (pointed by yellow arrows in Fig. 6b). The concave shape of the contact binding might be explained by CaCO_3_ precipitation while viscous pore water remained at the particle contacts.

**Figure 7:**
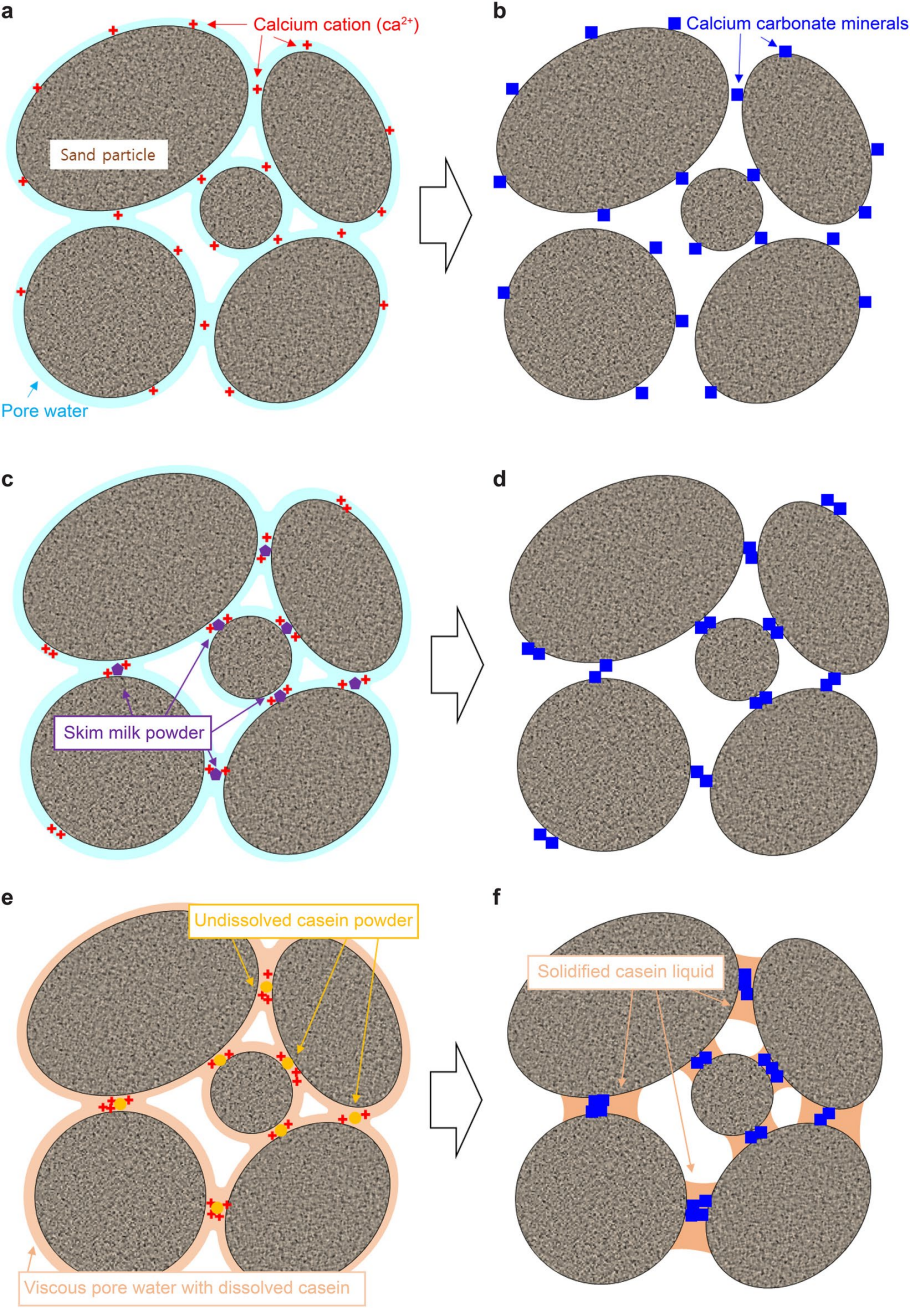
Schematic of the potential biocementation mechanisms via EICP only (**a** → **b**), EICP assisted using skim milk (**c** → **d**), and EICP assisted using casein (**e** → **f**).

Figure 7 is a schematic of the potential mechanisms of the EICP biocementation processes without any agents, with the skim-milk, and with casein. In the regular EICP process, the dissolved calcium ions that are evenly distributed through the pore water are attracted to the negatively charged sand particles (Fig. 7a). The calcium ions (red crosses in Fig. 7a) can precipitate with the dissolved carbonate ions released from urea hydrolysis, forming many small crystals of CaCO_3_ mineral over the entire domain (Fig. 7b). Under partially saturated conditions, the precipitation efficiency is increased and more precipitates form on the particle contacts. When skim-milk powder is added to the EICP process, multiple effects of the milk powder are expected. For instance, the precipitation rate is lowered because milk powder can reduce the number of active sites on the enzymes. In addition, skim-milk powder (indicated by the purple-edged pentagons in Fig. 7c) acts as a chelating agent that engages and aggregates the dissolved calcium cations in the pore water. CaCO_3_ precipitation is enhanced near the aggregated calcium-ion groups. Together with the low precipitation rate, these numerous calcium-ion sources increase the opportunity for crystal growth; consequently, large crystals may appear (Fig. 7d). Both patterns are easily observed in Fig. 6a. When casein is involved in the EICP treatment, it dissolves in the pore water under alkaline conditions while the urea hydrolysis proceeds. The dissolved casein increases the viscosity of pore water, hindering its migration and retaining it in the pore space. Undissolved casein powder attracts calcium ions through chelation, thereby creating larger crystals and enhancing the precipitation efficiency. The precipitation of dissolved casein can also contribute to the compressive strength of treated specimens by binding particles in company with the CaCO_3_ crystal groups (Fig. 7f). A very similar mechanism was previously reported for hydrogel-assisted EICP^29^. Such mechanisms prevail under partially saturated conditions. Fatehi et al.^30^ demonstrated a binding effect caused by casein itself with Iranian dune sand whose mean particle size was 0.16 mm. In their study, the UCS value reached 600 kPa with 1% of casein content. Considering less than 1% of casein content was included in the EICP treatment and the UCS strength of the EICP treated specimens without casein was around 1 MPa (Fig. 2), over 2 MPa of high UCS values of the casein-assisted EICP treated specimens can be justified with the described potential mechanisms in Fig. 7.

## Conclusions

In this study, a casein-assisted EICP treatment of Toyoura sand was experimentally optimized to enhance the compressive strength of the EICP. The main conclusions are summarized below.

- For a low theoretical CaCO_3_ precipitation content, higher UCS strengths were obtained from the casein-assisted EICP-treated specimens than from specimens reported in previous biocementation-related studies.
- As the casein content increased from 0 to 64.28 g/L in EICP compounds with constant urea/CaCl_2_ concentration (0.893 M/0.581 M), the mean UCS increased to 3.58 MPa.
- As the urea/CaCl_2_ concentration increased from 0.464 M/0.301 M to 1.036 M/0.673 M in the EICP compound with constant casein content (38.87 g/L), the mean UCS increased to 2.81 MPa.
- The casein-assisted EICP-treated specimens showed higher UCSs than the skim-milk-assisted EICP-treated specimens, regardless of assisting-agent concentration. This difference became more striking as the assisting-agent content increased.
- As casein was dissolved in the pore water while urea hydrolysis proceeded, the pore water became viscous and was retained in the pore space and at the particle-contact sites.
- In the specimens treated by EICP assisted by skim milk and casein, successful CaCO_3_ bindings between the sand particles were observed. The skin-milk specimens showed many large irregularly shaped precipitation patterns on the particle surfaces and contacts. The effectiveness of contact binding was improved in the casein specimens.
- In the casein-assisted EICP-treated specimens, precipitation of CaCO_3_ crystals along the viscous pore-water distribution might explain the concave-shaped binding observed at the particle contacts.

This study experimentally verified that casein is a more efficient assisting agent than skim milk in EICP treatment. The mechanical results were consolidated by SEM visualizations.

## Materials and methods

### Sand material

**Figure 8:**
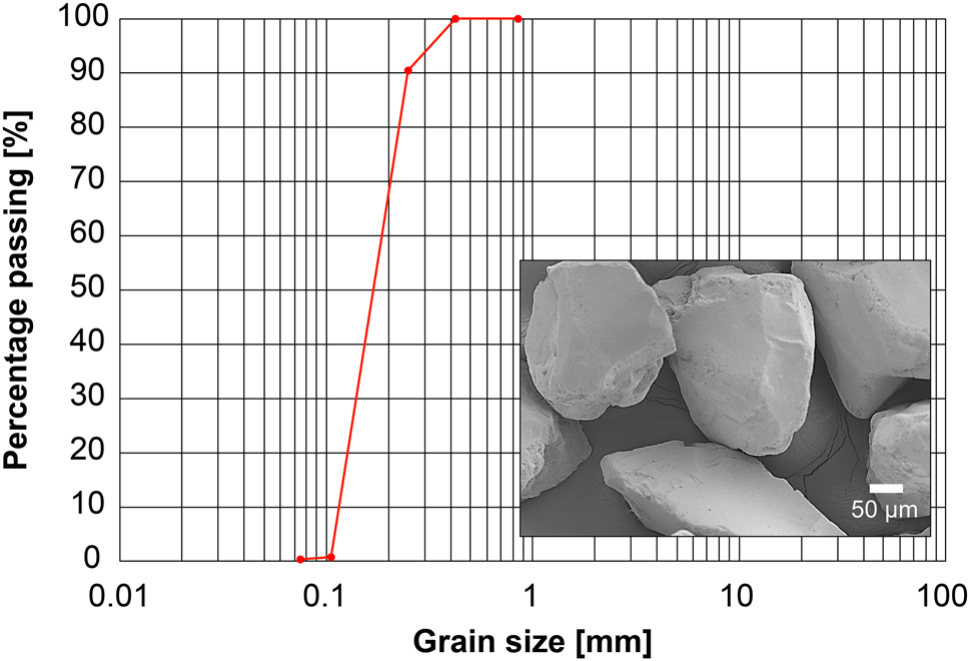
Particle-size distribution (red line with dots) and an SEM image (lower right corner) of the Toyoura sand.

All biocemented specimens included Toyoura sand, a silica granular material commonly used in geotechnical engineering studies in Japan^38–40^. The Toyoura sand had a mean grain size (*D*_50_) of 0.17 mm, a maximum void ratio (*e*_max_) of 0.978, a minimum void ratio (*e*_min_) of 0.597, and a specific gravity of solids (*G*_s_) of 2.64. A sieve analysis confirmed that this sand was poorly graded with a quite uniform grain size and low fine contents (Fig. 8). The grain shape ranged from angular to sub-angular (see the SEM image in the right-hand corner of Fig. 8). The initial characteristics of the target soil, e.g., the grain-size and pore-size distributions, relative density, and particle surface characteristics, determine permeability and fluid flow patterns which can affect the partial distribution of substances in the solution including enzymes, solutes, and assisting agents during the biocementation treatment. Therefore, the urea hydrolysis and precipitation processes of calcium carbonate mineral can be altered^3,41,42^. Toyoura sand was selected for this study because its physical uniformity is beneficial for minimizing potential sophisticated impacts on the biocementation process.

### Preparation of dry chemical compounds and EICP-biocemented specimen

The chemical compounds for the EICP treatment were prepared in dry powder form. The base EICP compounds were urea (CO(NH_2_)_2_, CAS 27-13-6, FUJIFILM Wako Pure Chemical Corporation, Osaka, Japan), calcium chloride (CaCl_2_, CAS 10043-52-4, FUJIFILM Wako Pure Chemical Corporation), and jack bean urease enzyme (CAS 9002-13-5, Junsei Chemical Co., Ltd., Tokyo, Japan). The concentrations of urea and CaCl_2_ were varied to meet the objective of each experimental study. The molar ratio of urea to CaCl_2_ was fixed at 1:0.65, which was empirically determined to maximize the calcium-ion consumption in a preliminary experiment. The efficiency of chemical consumption for calcium carbonate (CaCO_3_) precipitation through the EICP process should depend on the number of bicarbonate ions released from the urea hydrolysis, because CaCl_2_ is highly soluble in water. Adopting similar approaches, Almajed et al.^8^ and Martin et al.^43^ applied urea and CaCl_2_ at a molar ratio of 1:0.67 in lab-scale and mid-scale experiments of the EICP treatment. In these experiments, 2.5 g/L of urease enzyme with an activity of ~ 3500 U/g was included in all EICP compounds. Additional skim milk (198-10605, FUJIFILM Wako Pure Chemical Corporation) and casein protein powders (034-01508, FUJIFILM Wako Pure Chemical Corporation) were selectively added as assisting agents to the specific EICP compounds. Pure casein used in this study is extracted from milk. It is a white or slightly yellow small granule type. The manufacturer indicates that the melting point of the product is ~ 280 °C and it is practically insoluble in the water. The dissociation of casein protein has not been clearly investigated yet because it is a sophisticated phenomenon mutually affected by environmental factors such as ambient pH, temperature, and ion concentrations^44^. Ye and Harte^44^ stated that casein is dissociated at low and high pH and under high temperatures. Post et al.^45^ found that the solubility of casein is minimal at pH 2.0 to 5.0 in demineralized water and increases as pH increases from 5.0 and caseins are almost soluble at pH 10.0 to 11.0. Dried Toyoura sand (290 g) was mixed with the premixed EICP compound in a mortar mixer for 10 min. Next, 38.8 g of deionized (DI) water was added and thoroughly mixed. The sand, EICP compound, and DI water mixtures were placed into a 5-cm-diameter cylindrical mold in three lifts. Each lift was gently tamped 20 times, ensuring that the mixture reached a precise height of 9 cm. Under these packing conditions, the void ratio and water saturation degree of the specimens were 0.609 and 58%, respectively. After adding DI water, the mixing and packing procedure was completed in less than 5 min to promote all biocementation reactions under the stationary condition. The molds containing the mixtures were retained under different temperature-controlled conditions for curing and drying (see the following sections for the specific conditions of each experiment). The dried specimens were subjected to an unconfined UCS test at a constant axial strain rate of 0.9 mm/min (1% stretching of the sample height following the Japanese Geotechnical Society Standard, JGS 0511) to examine their mechanical performance enhancement.

### Optimization of the casein-assisted EICP biocementation

To investigate the effect of casein content on the EICP biocementation, Toyoura sand samples with fixed amounts of EICP components (0.893 M urea, 0.581 M CaCl_2_, and 2.6 g/L urea enzyme) were subjected to EICP treatments with various casein contents. The theoretical target of calcium carbonate precipitation from the EICP compound was 0.78% of the soil weight, assuming that all chemicals were dissolved and converted into CaCO_3_. When selecting the EICP concentration, we aimed to maximize the UCS strength (> 1 MPa) while minimizing the CaCO_3_ content (less than 1 wt% of soil) by a single treatment, as previously shown by Almajed et al.^8^.

Based on the fixed EICP compound, the casein content varied from 0 to 64.28 g/L in seven steps (0, 7.47, 25.41, 38.87, 46.34, 59.05, and 64.28 g/L). The specimens were prepared as described in the previous section, then cured at room temperature (20 °C) for 72 h and dried in the oven at 40 °C until their weight stabilized, indicating complete drying. After dislocating the mold, the biocemented specimens were subject to UCS testing. Four specimens with each casein content were prepared, giving 28 specimens in total. The specimen information and corresponding UCS results are presented in Table 1.

**Table 1:** Specimen information and corresponding UCS results of the casein-content optimization study with fixed EICP concentration.

EICP solution types	Specimen number	Targeting CaCO_3_ content	Basic EICP compound			Assisting agent		UCS (kPa)
			Urea (M)	CaCl_2_ (M)	Expected CaCO_3_ (g)	Skim milk (g/L)	Casein (g/L)	
EICP assisted by casein	A-1	0.78% of soil weight	0.893	0.581	2.255	–	–	1073
EICP assisted by casein	A-2	0.78% of soil weight	0.893	0.581	2.255	–	–	866
EICP assisted by casein	A-3	0.78% of soil weight	0.893	0.581	2.255	–	–	957
EICP assisted by casein	A-4	0.78% of soil weight	0.893	0.581	2.255	–	–	974
EICP assisted by casein	B-1	0.78% of soil weight	0.893	0.581	2.255	–	7.47	1031
EICP assisted by casein	B-2	0.78% of soil weight	0.893	0.581	2.255	–	7.47	979
EICP assisted by casein	B-3	0.78% of soil weight	0.893	0.581	2.255	–	7.47	1105
EICP assisted by casein	B-4	0.78% of soil weight	0.893	0.581	2.255	–	7.47	745
EICP assisted by casein	C-1	0.78% of soil weight	0.893	0.581	2.255	–	25.41	1891
EICP assisted by casein	C-2	0.78% of soil weight	0.893	0.581	2.255	–	25.41	1979
EICP assisted by casein	C-3	0.78% of soil weight	0.893	0.581	2.255	–	25.41	1935
EICP assisted by casein	C-4	0.78% of soil weight	0.893	0.581	2.255	–	25.41	1731
EICP assisted by casein	D-1	0.78% of soil weight	0.893	0.581	2.255		38.87	2168
EICP assisted by casein	D-2	0.78% of soil weight	0.893	0.581	2.255		38.87	1702
EICP assisted by casein	D-3	0.78% of soil weight	0.893	0.581	2.255		38.87	2852
EICP assisted by casein	D-4	0.78% of soil weight	0.893	0.581	2.255		38.87	2299
EICP assisted by casein	E-1	0.78% of soil weight	0.893	0.581	2.255	–	46.34	3094
EICP assisted by casein	E-2	0.78% of soil weight	0.893	0.581	2.255	–	46.34	3085
EICP assisted by casein	E-3	0.78% of soil weight	0.893	0.581	2.255	–	46.34	3424
EICP assisted by casein	E-4	0.78% of soil weight	0.893	0.581	2.255	–	46.34	2572
EICP assisted by casein	F-1	0.78% of soil weight	0.893	0.581	2.255		59.05	2960
EICP assisted by casein	F-2	0.78% of soil weight	0.893	0.581	2.255		59.05	2822
EICP assisted by casein	F-3	0.78% of soil weight	0.893	0.581	2.255		59.05	2955
EICP assisted by casein	F-4	0.78% of soil weight	0.893	0.581	2.255		59.05	3104
EICP assisted by casein	F-1	0.78% of soil weight	0.893	0.581	2.255		64.28	3142
EICP assisted by casein	F-2	0.78% of soil weight	0.893	0.581	2.255		64.28	3394
EICP assisted by casein	F-3	0.78% of soil weight	0.893	0.581	2.255		64.28	3814
EICP assisted by casein	F-4	0.78% of soil weight	0.893	0.581	2.255		64.28	3976

All specimens involved 2.6 g/L of urease enzyme.

The casein concentrations were determined as 0%, 0.1%, 0.34%, 0.52%, 0.62%, 0.79%, and 0.86% of soil weight.

All specimens were cured at 20 °C for 72 h and then dried at 40 °C.

### Optimal concentration of basic EICP compound with constant casein content

To optimize the concentration of the basic EICP compound, EICP-treated specimens containing different urea/CaCl_2_ concentrations and constant casein content were prepared. The casein content (38.87 g/L) had been optimized in a previous study. To meet the theoretically targeted CaCO_3_ contents (0.4%–0.9% of soil weight), the urea/CaCl_2_ concentration was varied as 0.464 M/0.301 M, 0.679 M/0.441 M, 0.893 M/0.581 M, and 0.1560 M/1.014 M. Four specimens were prepared for each concentration of EICP compound (16 specimens in total) and subjected to USC measurement. The specimen information and corresponding UCS results are organized in Table 2. The sampling, curing, and drying methodologies were unchanged from the previous study.

**Table 2:** Specimen information and corresponding UCS results of the EICP-compound optimization study with fixed casein content.

EICP solution types	Specimen number	Targeting CaCO_3_ content	Basic EICP compound			Assisting agent		UCS (kPa)
			Urea (M)	CaCl_2_ (M)	Expected CaCO_3_ (g)	Skim milk (g/L)	Casein (g/L)	
EICP assisted by casein	A-1	0.40% of soil weight	0.464	0.301	1.172	–	38.87	613
EICP assisted by casein	A-2	0.40% of soil weight	0.464	0.301	1.172	–	38.87	891
EICP assisted by casein	A-3	0.40% of soil weight	0.464	0.301	1.172	–	38.87	Fail
EICP assisted by casein	A-4	0.40% of soil weight	0.464	0.301	1.172	–	38.87	Fail
EICP assisted by casein	B-1	0.59% of soil weight	0.679	0.441	1.714	–	38.87	2018
EICP assisted by casein	B-2	0.59% of soil weight	0.679	0.441	1.714	–	38.87	2023
EICP assisted by casein	B-3	0.59% of soil weight	0.679	0.441	1.714	–	38.87	2067
EICP assisted by casein	B-4	0.59% of soil weight	0.679	0.441	1.714	–	38.87	1778
EICP assisted by casein	C-1	0.78% of soil weight	0.893	0.581	2.255	–	38.87	2168
EICP assisted by casein	C-2	0.78% of soil weight	0.893	0.581	2.255	–	38.87	1702
EICP assisted by casein	C-3	0.78% of soil weight	0.893	0.581	2.255	–	38.87	2852
EICP assisted by casein	C-4	0.78% of soil weight	0.893	0.581	2.255	–	38.87	2299
EICP assisted by casein	D-1	0.90% of soil weight	1.036	0.673	2.615		38.87	3490
EICP assisted by casein	D-2	0.90% of soil weight	1.036	0.673	2.615		38.87	2921
EICP assisted by casein	D-3	0.90% of soil weight	1.036	0.673	2.615		38.87	2016
EICP assisted by casein	D-4	0.90% of soil weight	1.036	0.673	2.615		38.87	Fail

All specimens involved 2.6 g/L of urease enzyme.

The urea/calcium chloride concentrations were determined to meet the theoretically targeted calcium carbonate precipitation (0.4, 0.6, 0.8, and 0.9 wt% of soil).

All specimens were cured at 25 °C for 72 h and then dried at 40 °C.

### Curing and drying environments of the casein-EICP treatment

In many of the related studies, the EICP-treated specimens were simply cured at a controlled temperature (typically, at room temperature)^8,46^. Approximately 80% of casein is protein, which can be extracted from milk and is generally insoluble in neutral-pH water but dissolves in alkaline environments^47^. We assumed that casein is dissolved when the ambient pore water becomes alkaline after urea hydrolysis. To verify this assumption, we established three distinguished specimen-curing strategies: (1) immediate oven-drying of the specimens at 105 °C, (2) curing at room temperature (20 °C) for 72 h followed by drying at 105 °C, and (3) curing at 20 °C followed by drying at 40 °C. The EICP contents of all specimens (0.893 M urea, 0.581 M CaCl_2_, 2.6 g/L urea enzyme, and 38.87 g/L casein) had been optimized in a previous study. Eight specimens were prepared: two specimens each in cases A and B (see below for explanation) and four specimens in Case C. The specimens were treated, cured, and dried, then subjected to UCS tests. The specimen information and corresponding UCS measurements are presented in Table 3.

**Table 3:** Specimen information for EICP treatment and corresponding UCS results of the optimization study of curing and drying strategies.

EICP solution types	Specimen number	Targeting CaCO_3_ content	Basic EICP compound			Assisting agent		UCS (kPa)
			Urea (M)	CaCl_2_ (M)	Expected CaCO_3_ (g)	Skim milk (g/L)	Casein (g/L)	
EICP assisted by casein	A-1	0.78% of soil weight	0.893	0.581	2.255	–	38.87	278
EICP assisted by casein	A-2	0.78% of soil weight	0.893	0.581	2.255	–	38.87	208
EICP assisted by casein	B-1	0.78% of soil weight	0.893	0.581	2.255	–	38.87	1708
EICP assisted by casein	B-2	0.78% of soil weight	0.893	0.581	2.255	–	38.87	1558
EICP assisted by casein	C-1	0.78% of soil weight	0.893	0.581	2.255	–	38.87	2168
EICP assisted by casein	C-2	0.78% of soil weight	0.893	0.581	2.255	–	38.87	1702
EICP assisted by casein	C-3	0.78% of soil weight	0.893	0.581	2.255	–	38.87	2852
EICP assisted by casein	C-4	0.78% of soil weight	0.893	0.581	2.255	–	38.87	2299

All specimens involves 2.6 g/L of urease enzyme.

Specimens of case A were dried at 105 °C after the mixing process.

Specimens of case B were cured at 25 °C for 72 h and then dried at 105 °C.

Specimens of case C were cured at 25 °C for 72 h and then dried at 40 °C.

In Strategy A, the urea hydrolysis was restricted by high temperature, ensuring insufficient casein dissolution. In Strategies B and C, the EICP process was continued for 72 h and longer, respectively.

### Comparison of biocemented specimens assisted by skim milk and casein protein

To verify whether casein in milk powder can increase the size of CaCl_2_ crystals in the EICP biocementation process, we compared the UCS results of the EICP-treated specimens assisted by skim milk and casein at low and high concentrations. The base EICP compounds were fixed (0.893 M urea, 0.581 M CaCl_2_, 2.6 g/L urease enzyme) to achieve a theoretical precipitation level of 0.78 wt% of soil. Low (3.89 g/L) and high (38.87 g/L) contents of skim milk and casein were then added to the dry EICP compounds before dry-mixing with Toyoura sand. Note that the high concentration was 10 times that of the low concentration. The water mixing and specimen preparation were described in the previous experiments. After inserting the final mixture into the mold, the specimens were cured and dried following Strategy C in the previous section. Three specimens were prepared for each case (12 specimens in total) and subjected to USC tests. The specimen information and corresponding UCS results are listed in Table 4. Selected intact chunks of the cemented specimens were investigated by SEM (JSM-6010PLUS/LA, JEOL Ltd., province, country), which revealed the morphology of the precipitated CaCO_3_ crystals and the microscopic precipitation patterns. For SEM imaging, the sample surface was coated with a thin uniform layer of platinum–palladium particles through an evaporation process to prevent charging of the sample surface and to increase the number of generated secondary electrons, thus providing a clean image.

**Table 4:** Specimen information for EICP treatment and corresponding UCS results for the optimization study of curing and drying strategies.

EICP solution types	Specimen number	Targeting CaCO_3_ content	Basic EICP compound			Assisting agent		UCS (kPa)
			Urea (M)	CaCl_2_ (M)	Expected CaCO_3_ (g)	Skim milk (g/L)	Casein (g/L)	
EICP assisted by skim milk	EML-1	0.78% of soil weight	0.893	0.581	2.255	3.89	–	884
EICP assisted by skim milk	EML-2	0.78% of soil weight	0.893	0.581	2.255	3.89	–	773
EICP assisted by skim milk	EML-3	0.78% of soil weight	0.893	0.581	2.255	3.89	–	721
EICP assisted by casein	ECL-1	0.78% of soil weight	0.893	0.581	2.255	–	3.89	1067.5
EICP assisted by casein	ECL-2	0.78% of soil weight	0.893	0.581	2.255	–	3.89	729.8
EICP assisted by casein	ECL-3	0.78% of soil weight	0.893	0.581	2.255	–	3.89	Fail
EICP assisted by skim milk	EMH-1	0.78% of soil weight	0.893	0.581	2.255	38.87	–	1387
EICP assisted by skim milk	EMH-2	0.78% of soil weight	0.893	0.581	2.255	38.87	–	1235
EICP assisted by skim milk	EMH-3	0.78% of soil weight	0.893	0.581	2.255	38.87	–	1132
EICP assisted by casein	ECH-1	0.78% of soil weight	0.893	0.581	2.255	–	38.87	2168
EICP assisted by casein	ECH-2	0.78% of soil weight	0.893	0.581	2.255	–	38.87	2299
EICP assisted by casein	ECH-3	0.78% of soil weight	0.893	0.581	2.255	–	38.87	2852

All specimens involves 2.6 g/L of urease enzyme.

The skim milk and casein contents were determined as 0.052% and 0.52% of soil weight for low and high concentrations correspondingly.

## Data Availability

All data generated or analyzed during this study are included in this published article.
